# Supplementary material for: Stimulating the hippocampal posterior-medial network enhances task-dependent connectivity and memory
Source: eLife. 2019 Nov 14;8:e49458. doi: 10.7554/eLife.49458 (PMC6855798; doi:10.7554/eLife.49458)
Supplement: Supplementary file 1. [file elife-49458-supp1.docx]

**Supplementary File 1: PMN and ATN effect tables**

| **PMN** | | | | | | | | | | |
| --- | --- | --- | --- | --- | --- | --- | --- | --- | --- | --- |
| **Effect** | **Beta Estimate** | | **Std. Error** | | **df** | | **t** | | **p** | |
| (Intercept) | 0.768 | | 0.089 | | 112.100 | | 8.627 | | 0.000 | |
| State | 0.054 | | 0.029 | | 91.010 | | 1.846 | | 0.068 | |
| Condition | 0.035 | | 0.029 | | 89.610 | | 1.197 | | 0.234 | |
| Target | 0.016 | | 0.040 | | 70.880 | | 0.399 | | 0.691 | |
| State x Condition | -0.063 | | 0.041 | | 89.730 | | -1.553 | | 0.124 | |
| State x Target | -0.066 | | 0.041 | | 89.550 | | -1.622 | | 0.108 | |
| Condition x Target | -0.062 | | 0.041 | | 90.180 | | -1.505 | | 0.136 | |
| State x Condition x Target | 0.161 | | 0.058 | | 89.600 | | 2.800 | | 0.006* | |
| **ATN** | | | | | | | | | | |
| **Effect** | | **Beta Estimate** | | **Std. Error** | | **df** | | **t** | | **p** |
| (Intercept) | | 0.522 | | 0.077 | | 113.000 | | 6.810 | | 0.000 |
| State | | 0.012 | | 0.025 | | 90.770 | | 0.488 | | 0.627 |
| Condition | | 0.037 | | 0.025 | | 89.390 | | 1.497 | | 0.138 |
| Target | | -0.013 | | 0.035 | | 68.670 | | -0.372 | | 0.711 |
| State x Condition | | -0.041 | | 0.035 | | 89.510 | | -1.176 | | 0.243 |
| State x Target | | -0.041 | | 0.035 | | 89.330 | | -1.175 | | 0.243 |
| Condition x Target | | -0.019 | | 0.035 | | 89.960 | | -0.539 | | 0.592 |
| State x Condition x Target | | 0.073 | | 0.049 | | 89.380 | | 1.482 | | 0.142 |
