# Supplementary material for: Stimulating the hippocampal posterior-medial network enhances task-dependent connectivity and memory
Source: eLife. 2019 Nov 14;8:e49458. doi: 10.7554/eLife.49458 (PMC6855798; doi:10.7554/eLife.49458)
Supplement: Supplementary file 2. — Region labels from Eickhoff-Zilles macro labels from N27 in MNI space. Note that ‘calcarine gyrus’ refers to the area surrounding the calcarine sulcus, including the precuneus and lingual gyrus. [file elife-49458-supp2.docx]

**Supplementary File 2: PMN and ATN ROI abbreviations**

| **PMN** | | | | |
| --- | --- | --- | --- | --- |
|  | **Region** | **x** | **y** | **z** |
| Thal | Thalamus | -21 | 28 | 8 |
| SMG | Supramarginal Gyrus | -49 | 47 | 26 |
| SOG | Superior Occipital Gyrus | 15 | 92 | 17 |
| Cun | Cuneus | -17 | 66 | 21 |
| CalG | Calcarine Gyrus | -13 | 68 | 7 |
| LiG | Lingual Gyrus | 13 | 47 | -4 |
| CalG | Calcarine Gyrus | -13 | 82 | 2 |
| LiG | Lingual Gyrus | 2 | 73 | -2 |
| LiG | Lingual Gyrus | -17 | 43 | -2 |
| Prec | Precuneus | -17 | 51 | 32 |
| Prec | Precuneus | 2 | 59 | 30 |
| Prec | Precuneus | 11 | 50 | 36 |
| HC | Hippocampus | 19 | 27 | -5 |
| HC | Hippocampus | -19 | 28 | -3 |
| Prec | Precuneus | 7 | 48 | 13 |
| Prec | Precuneus | -8 | 44 | 15 |
| CalG | Calcarine Gyrus | 13 | 58 | 16 |
| CalG | Calcarine Gyrus | -6 | 55 | 13 |
| MOG | Middle Occipital Gyrus | 35 | 79 | 24 |
| **ATN** | | | | |
|  | **Region** | **x** | **y** | **z** |
| SMeG | Superior Medial Gyrus | 2 | -54 | 37 |
| ITG | Inferior Temporal Gyrus | 39 | 13 | -23 |
| ITG | Inferior Temporal Gyrus | -60 | 13 | -22 |
| MTG | Middle Temporal Gyrus | 60 | 33 | -7 |
| PHG | Parahippocampal Gyrus | -21 | 5 | -19 |
| MTG | Middle Temporal Gyrus | -66 | 35 | -7 |
| SFG | Superior Frontal Gyrus | 22 | -55 | 29 |
| ITG | Inferior Temporal Gyrus | 41 | -3 | -33 |
| PHG | Parahippocampal Gyrus | 22 | 11 | -23 |
| FuG | Fusiform Gyrus | -28 | 11 | -29 |
| SFG | Superior Frontal Gyrus | -17 | -53 | 28 |
| FuG | Fusiform Gyrus | -38 | 16 | -22 |
| ITG | Inferior Temporal Gyrus | -51 | 3 | -24 |
| IFG | Inferior Frontal Gyrus | -23 | -10 | -16 |
