# Supplementary material for: Stimulating the hippocampal posterior-medial network enhances task-dependent connectivity and memory
Source: eLife. 2019 Nov 14;8:e49458. doi: 10.7554/eLife.49458 (PMC6855798; doi:10.7554/eLife.49458)
Supplement: Supplementary file 3. — Region labels from Eickhoff-Zilles macro labels from N27 in MNI space. Note that ‘calcarine gyrus’ refers to the area surrounding the calcarine sulcus, including the precuneus and lingual gyrus. [file elife-49458-supp3.docx]

**Supplementary File 3: Findings of the demand-selective connectivity analysis and their corresponding drivers for PMN-targeted stimulation.**

| **Cluster Peak (RAI)** | | | **Region** |
| --- | --- | --- | --- |
| **x** | **y** | **z** |  |
| **-21** | **65** | **14** | **Calcarine Gyrus** |
| -9 | 59 | 54 | Precuneus |
| 49 | -9 | -8 | Superior Temporal Gyrus/Temporal Pole |
| -1 | -37 | 36 | Superior Medial Gyrus |
| -49 | -15 | 28 | Inferior Frontal Gyrus |
| -29 | 3 | 58 | Superior Frontal Gyrus |
| -21 | -23 | 42 | Middle Frontal Gyrus |
| -55 | 49 | 2 | Middle Temporal Gyrus |
| -7 | -17 | 36 | Middle Cingulate Gyrus |
| **9** | **45** | **10** | **PCC/Precuneus** |
| -37 | 65 | 38 | Angular Gyrus |
| -45 | -37 | 2 | Inferior Frontal Gyrus |
| 47 | -7 | 32 | Precentral Gyrus |
| -43 | -7 | 46 | Middle Frontal Gyrus |
| **39** | **9** | **12** | **Insula** |
| 53 | -11 | -2 | Superior Temporal Gyrus/Temporal Pole |
| -7 | -35 | 32 | Anterior Cingulate Cortex |
| -59 | 33 | -12 | Inferior Temporal Gyrus |
| 47 | 55 | -6 | Inferior Temporal Gyrus |
| -35 | -29 | 14 | Inferior Frontal Gyrus |
| -17 | 57 | 12 | Calcarine Gyrus |
| -19 | 65 | 48 | Superior Parietal Lobule |
| 17 | 59 | 10 | Calcarine Gyrus |
| -5 | 15 | 54 | SMA |
| 43 | 19 | 44 | Postcentral Gyrus |
| -7 | -47 | -8 | Rectal Gyrus |
| **-19** | **35** | **-16** | **Cerebellum** |
| 11 | 73 | 24 | Cuneus |
| -15 | 53 | -8 | Lingual Gyrus |
| -23 | 77 | -18 | Cerebellum |
| -39 | 61 | -22 | Cerebellum |
| 47 | 47 | 14 | Middle Temporal Gyrus |
| 39 | -3 | 40 | Precentral Gyrus |
| -31 | 63 | -36 | Cerebellum |
| 17 | 57 | 38 | Superior Parietal Lobule |
| 55 | 43 | 42 | Inferior Parietal Lobule |
| 33 | -33 | 4 | Inferior Frontal Gyrus |
| 63 | 37 | 14 | Superior Temporal Gyrus |
| -3 | -21 | 28 | Anterior Cingulate Cortex |
| 17 | -31 | 32 | Superior Frontal Gyrus |
| 43 | -1 | 40 | Precentral Gyrus |
| -37 | 33 | 56 | Postcentral Gyrus |
| **9** | **-31** | **0** | **ACC** |
| -49 | -17 | 8 | Inferior Frontal Gyrus |
| 7 | -27 | 28 | Anterior Cingulate Cortex |
| 39 | -37 | 12 | Inferior Frontal Gyrus |
| 57 | 53 | 18 | Middle Temporal Gyrus |
| 27 | -47 | 4 | Middle Orbital Gyrus |
| 41 | 13 | 54 | Precentral Gyrus |
| 9 | 73 | -34 | Cerebellum |
| 1 | -19 | 48 | Superior Medial Gyrus |
| -1 | -47 | 28 | Superior Medial Gyrus |
| -19 | -9 | 4 | Putamen |
| 51 | -3 | 24 | Inferior Frontal Gyrus |
| 47 | 33 | 22 | Superior Temporal Gyrus |
| -3 | 29 | 26 | Middle Cingulate Gyrus |
| 23 | 59 | 16 | Calcarine Gyrus |
| -19 | -27 | 32 | Superior Frontal Gyrus |
| 15 | -11 | 60 | Superior Frontal Gyrus |
| -43 | 21 | 44 | Postcentral Gyrus |
| 17 | 9 | 62 | SMA |
| -5 | 71 | 12 | Calcarine Gyrus |
| **9** | **39** | **-16** | **Cerebellum** |
| 17 | 51 | 2 | Lingual Gyrus |
| -11 | 57 | -4 | Lingual Gyrus |
| 7 | 81 | 14 | Cuneus |
| 11 | 73 | -4 | Lingual Gyrus |
| -31 | 47 | 44 | Inferior Parietal Lobule |
| -47 | 39 | 52 | Superior Parietal Lobule |
| 47 | -15 | 10 | Inferior Frontal Gyrus |
| 49 | 57 | 30 | Angular Gyrus |
| **21** | **15** | **-22** | **MTL** |
| -17 | 45 | 32 | Middle Cingulate Gyrus |
| 41 | 1 | 44 | Precentral Gyrus |
| 53 | -5 | 22 | Inferior Frontal Gyrus |
| 47 | 45 | 6 | Middle Temporal Gyrus |
| 1 | 1 | 58 | SMA |
| **-47** | **13** | **8** | **Heschls Gyrus** |
| -7 | 45 | 10 | Precuneus |
| 5 | 51 | 56 | Precuneus |
| 43 | 55 | 0 | Middle Temporal Gyrus |
| 15 | 63 | -8 | Lingual Gyrus |
| 5 | -3 | 44 | SMA |
| -5 | -21 | 42 | Superior Medial Gyrus |
| 27 | 13 | 52 | Precentral Gyrus |
| -21 | 5 | 56 | Superior Frontal Gyrus |
| 51 | -11 | 38 | Middle Frontal Gyrus |
| -37 | 75 | -8 | Inferior Occipital Gyrus |
| 55 | -13 | 2 | Superior Temporal Gyrus/Temporal Pole |
| 51 | 67 | -6 | Inferior Occipital Gyrus |
| **19** | **41** | **16** | **Caudate** |
| -25 | 65 | -22 | Cerebellum |
| 7 | 49 | 58 | Precuneus |
| 49 | 39 | 4 | Middle Temporal Gyrus |
| 41 | 21 | -4 | Superior Temporal Gyrus |
| 51 | 13 | 4 | Superior Temporal Gyrus |
| **-35** | **19** | **46** | **Precentral Gyrus** |
| 3 | 29 | -34 | Brainstem |
| 31 | 67 | -4 | Inferior Occipital Gyrus |
| -25 | 79 | -16 | Fusiform Gyrus |
| 5 | 93 | 10 | Cuneus |
| 1 | 61 | 36 | Precuneus |
| 23 | -9 | 46 | Middle Frontal Gyrus |
| 1 | 53 | 56 | Precuneus |
| **-49** | **23** | **18** | **Insula** |
| 43 | 49 | 44 | Inferior Parietal Lobule |
| -9 | 41 | 44 | Middle Cingulate Gyrus |
| **49** | **-5** | **-6** | **STG/Temporal Pole** |
| -7 | 25 | 42 | Middle Cingulate Gyrus |
| 43 | -37 | 14 | Inferior Frontal Gyrus |
| -15 | 71 | 10 | Calcarine Gyrus |
| 25 | 63 | -12 | Fusiform Gyrus |
| 17 | 33 | 56 | Postcentral Gyrus |
| -33 | 19 | -10 | Hippocampus |
| -29 | 63 | 18 | Middle Occipital Gyrus |
| -43 | 5 | 48 | Precentral Gyrus |
| **9** | **59** | **28** | **Precuneus** |
| 39 | -1 | 36 | Precentral Gyrus |
| 37 | 69 | -20 | Cerebellum |
| -13 | 71 | -22 | Cerebellum |
| 1 | 39 | 6 | Cerebellum |
| **33** | **19** | **-18** | **Parahippocampal Gyrus** |
| -7 | -17 | 44 | Superior Medial Gyrus |
| 13 | 77 | -22 | Cerebellum |
| 43 | 61 | 32 | Angular Gyrus |
| -63 | 17 | -6 | Middle Temporal Gyrus |
| 57 | 27 | 2 | Middle Temporal Gyrus |
| -5 | -31 | 30 | Anterior Cingulate Cortex |
| -47 | 61 | -18 | Fusiform Gyrus |
| -17 | 19 | -6 | Hippocampus |
| -53 | -7 | 34 | Precentral Gyrus |
| -9 | 83 | -2 | Calcarine Gyrus |
| -19 | 89 | 2 | Superior Occipital Gyrus |
| -37 | -43 | 12 | Middle Frontal Gyrus |
| 21 | 91 | 4 | Middle Occipital Gyrus |
| 31 | 5 | 54 | Precentral Gyrus |
| -23 | -41 | -10 | Middle Orbital Gyrus |
| 7 | 55 | 22 | Precuneus |
| -3 | -35 | 8 | Anterior Cingulate Cortex |
| 5 | 51 | 54 | Precuneus |
| 21 | 85 | 26 | Superior Occipital Gyrus |
| -13 | 15 | 46 | Middle Cingulate Gyrus |
| -19 | 63 | -8 | Lingual Gyrus |
| -1 | 41 | 28 | Posterior Cingulate Cortex |
| -19 | 31 | 48 | Paracentral Lobule |
| 23 | 5 | 64 | Superior Frontal Gyrus |
| 43 | 45 | 44 | Inferior Parietal Lobule |
| -9 | 69 | 50 | Precuneus |
| -45 | -29 | 26 | Middle Frontal Gyrus |
| **1** | **65** | **44** | **Precuneus** |
| -43 | -7 | 22 | Inferior Frontal Gyrus |
| -41 | 65 | -8 | Inferior Temporal Gyrus |
| -37 | 79 | -8 | Inferior Occipital Gyrus |
| -7 | 49 | 48 | Precuneus |
| 5 | 59 | 6 | Lingual Gyrus |
| 47 | 55 | -8 | Inferior Temporal Gyrus |
| 15 | 73 | -16 | Cerebellum |
| **17** | **71** | **18** | **SOG** |
| -51 | 27 | 38 | Supramarginal Gyrus |
| 35 | 77 | -4 | Inferior Occipital Gyrus |
| -45 | -3 | 26 | Inferior Frontal Gyrus |
| -5 | 75 | 8 | Calcarine Gyrus |
| -27 | 79 | 26 | Superior Occipital Gyrus |
| 25 | -29 | -4 | Inferior Frontal Gyrus |
| -47 | 55 | 38 | Inferior Parietal Lobule |
| **-55** | **41** | **20** | **STG** |
| -33 | -3 | 54 | Middle Frontal Gyrus |
| 35 | 47 | 40 | Inferior Parietal Lobule |
| **1** | **65** | **30** | **Precuneus** |
| -3 | 47 | -14 | Cerebellum |
| 39 | -39 | 18 | Inferior Frontal Gyrus |
