# Supplementary material for: Stimulating the hippocampal posterior-medial network enhances task-dependent connectivity and memory
Source: eLife. 2019 Nov 14;8:e49458. doi: 10.7554/eLife.49458 (PMC6855798; doi:10.7554/eLife.49458)
Supplement: Supplementary file 4. — Region labels from Eickhoff-Zilles macro labels from N27 in MNI space. Note that ‘calcarine gyrus’ refers to the area surrounding the calcarine sulcus, including the precuneus and lingual gyrus. [file elife-49458-supp4.docx]

**Supplementary File 4: Findings of the demand-selective connectivity analysis and their corresponding drivers for PFC-targeted stimulation.**

| **Cluster Peak (RAI)** | | | **Region** |
| --- | --- | --- | --- |
| **x** | **y** | **z** |  |
| **-7** | **27** | **44** | **Middle Cingulate Cortex** |
| 41 | 7 | 42 | Precentral Gyrus |
| -37 | -19 | -16 | Superior Temporal Gyrus/Temporal Pole |
| 59 | -5 | 18 | Inferior Frontal Gyrus |
| -1 | -53 | 10 | Superior Medial Gyrus |
| 55 | 15 | 12 | Superior Temporal Gyrus |
| 33 | 59 | 34 | Angular Gyrus |
| -35 | 47 | -18 | Fusiform Gyrus |
| -45 | 15 | 52 | Precentral Gyrus |
| -1 | 65 | 20 | Cuneus |
| 45 | -19 | -12 | Superior Temporal Gyrus/Temporal Pole |
| -25 | 19 | 66 | Precentral Gyrus |
| -53 | 9 | 32 | Postcentral Gyrus |
| 39 | 3 | 6 | Insula |
| -15 | 81 | -6 | Lingual Gyrus |
| -63 | 33 | 4 | Middle Temporal Gyrus |
| 23 | -13 | 40 | Middle Frontal Gyrus |
| 11 | 53 | -14 | Cerebellum |
| -21 | -19 | 44 | Middle Frontal Gyrus |
| 57 | 3 | -6 | Superior Temporal Gyrus |
| 47 | 47 | 26 | Supramarginal Gyrus |
| -37 | -35 | -2 | Inferior Frontal Gyrus |
| **-5** | **61** | **-28** | **Cerebellar Vermis** |
| -15 | 35 | 40 | Middle Cingulate Gyrus |
| 1 | 67 | 6 | Lingual Gyrus |
| -15 | -21 | 44 | Superior Frontal Gyrus |
| 17 | -55 | 30 | Superior Frontal Gyrus |
| -49 | 45 | 22 | Supramarginal Gyrus |
| 23 | 41 | 62 | Postcentral Gyrus |
| -9 | -13 | 60 | SMA |
| -3 | -45 | -4 | Middle Orbital Gyrus |
| 57 | 61 | 16 | Middle Temporal Gyrus |
| -55 | 7 | -6 | Superior Temporal Gyrus |
| **25** | **-33** | **0** | **Inferior Frontal Gyrus** |
| 55 | 47 | 30 | Supramarginal Gyrus |
| **-25** | **-11** | **-2** | **Putamen** |
| -21 | 77 | -28 | Cerebellum |
| -23 | 39 | 52 | Postcentral Gyrus |
| -11 | -59 | 30 | Superior Medial Gyrus |
| 37 | -21 | 0 | Inferior Frontal Gyrus |
| -3 | -53 | 16 | Superior Medial Gyrus |
| -53 | -7 | -2 | Superior Temporal Gyrus/Temporal Pole |
| -7 | -5 | 12 | Caudate Nucleus |
| 15 | 63 | -32 | Cerebellum |
| -27 | 1 | 58 | Superior Frontal Gyrus |
| -17 | 79 | 0 | Calcarine Gyrus |
| -39 | -17 | -24 | Medial Temporal Pole |
| 35 | -19 | 46 | Middle Frontal Gyrus |
| 37 | -13 | -26 | Medial Temporal Pole |
| 13 | 1 | 18 | Caudate Nucleus |
| 49 | 25 | 28 | Supramarginal Gyrus |
| -45 | 45 | -6 | Inferior Temporal Gyrus |
| -53 | 15 | 26 | Supramarginal Gyrus |
| 49 | 1 | 38 | Precentral Gyrus |
| -39 | 63 | 30 | Angular Gyrus |
| 31 | 69 | 46 | Superior Parietal Lobule |
| 59 | 31 | -10 | Middle Temporal Gyrus |
| 39 | 55 | 28 | Angular Gyrus |
| -33 | 33 | 50 | Postcentral Gyrus |
| -29 | 17 | 56 | Superior Frontal Gyrus |
| 9 | 9 | 52 | SMA |
| 27 | 49 | -22 | Cerebellum |
| -53 | 1 | 38 | Precentral Gyrus |
| 17 | -7 | 56 | Superior Frontal Gyrus |
| -31 | 23 | 30 | Postcentral Gyrus |
| 43 | 49 | -18 | Fusiform Gyrus |
| 43 | -23 | -4 | Inferior Frontal Gyrus |
| **-13** | **-43** | **-4** | **Middle Occipital Gyrus** |
| 27 | 41 | -18 | Fusiform Gyrus |
| -47 | 55 | -8 | Inferior Temporal Gyrus |
| -21 | -1 | 10 | Putamen |
| -27 | -45 | 26 | Middle Frontal Gyrus |
| -39 | 61 | -24 | Cerebellum |
| -23 | 1 | 8 | Putamen |
| -1 | 7 | 38 | Middle Cingulate Gyrus |
| 55 | 21 | 22 | Postcentral Gyrus |
| -7 | 59 | 48 | Precuneus |
| 15 | 63 | 52 | Superior Parietal Lobule |
| **-31** | **77** | **22** | **ACC/Orbital Gyrus** |
| 51 | -5 | 14 | Inferior Frontal Gyrus |
| 51 | 63 | 8 | Middle Temporal Gyrus |
| -61 | 37 | 26 | Supramarginal Gyrus |
| -7 | 25 | 2 | Thalamus |
| 13 | -53 | 2 | Superior Orbital Gyrus |
| -5 | -25 | 38 | Middle Cingulate Gyrus |
| 3 | 43 | 50 | Precuneus |
| **-13** | **-43** | **24** | **Insula/Inferior Frontal Gyrus** |
| -29 | -15 | 46 | Middle Frontal Gyrus |
| **35** | **-21** | **4** | **ACC** |
| 49 | 7 | 28 | Precentral Gyrus |
| 29 | -27 | -10 | Inferior Frontal Gyrus |
| 7 | -25 | 14 | Anterior Cingulate Cortex |
| -49 | 51 | -22 | Inferior Temporal Gyrus |
| 35 | 9 | 0 | Insula |
| 39 | -7 | 36 | Middle Frontal Gyrus |
| 47 | 53 | -16 | Inferior Temporal Gyrus |
| 33 | 41 | 12 | Superior Temporal Gyrus |
| 5 | 31 | 42 | Middle Cingulate Gyrus |
| **-23** | **39** | **-4** | **Parahippocampal Gyrus** |
| -27 | -37 | -4 | Middle Orbital Gyrus |
| -7 | 51 | 58 | Precuneus |
| -37 | 55 | -38 | Cerebellum |
| **33** | **3** | **-22** | **Parahippocampal Gyrus** |
| -43 | -29 | 28 | Inferior Frontal Gyrus |
| -13 | -45 | -8 | Superior Orbital Gyrus |
| 45 | 65 | 32 | Angular Gyrus |
| 23 | 73 | 44 | Superior Parietal Lobule |
| -27 | -55 | 10 | Middle Frontal Gyrus |
| 25 | -21 | 50 | Middle Frontal Gyrus |
| -31 | 27 | 16 | Heschls Gyrus |
| -67 | 27 | 2 | Middle Temporal Gyrus |
| 33 | 11 | -10 | Hippocampus |
| -47 | 13 | 26 | Postcentral Gyrus |
| 15 | 49 | -20 | Cerebellum |
| 9 | 33 | 4 | Hippocampus |
| -63 | 17 | -8 | Middle Temporal Gyrus |
| -9 | 21 | 42 | Middle Cingulate Gyrus |
| -33 | -7 | 36 | Middle Frontal Gyrus |
| 7 | -25 | 32 | Superior Medial Gyrus |
| -29 | 13 | 8 | Putamen |
| -59 | -1 | 12 | Rolandic Operculum |
| 1 | 11 | 66 | SMA |
| 51 | -11 | 16 | Inferior Frontal Gyrus |
| -53 | 33 | 6 | Middle Temporal Gyrus |
| -49 | 27 | 22 | Rolandic Operculum |
| -51 | 15 | 28 | Supramarginal Gyrus |
| -33 | -35 | 20 | Middle Frontal Gyrus |
| -37 | 59 | 32 | Angular Gyrus |
| **-29** | **59** | **32** | **ACC** |
| 39 | -23 | -6 | Inferior Frontal Gyrus |
| 57 | 41 | 26 | Supramarginal Gyrus |
| 7 | 69 | 46 | Precuneus |
| -1 | -43 | 40 | Superior Medial Gyrus |
| **-35** | **-39** | **2** | **Inferior Frontal Gyrus** |
| 59 | 5 | -2 | Superior Temporal Gyrus |
| 1 | -31 | 54 | Superior Medial Gyrus |
| -47 | 43 | -2 | Middle Temporal Gyrus |
| 45 | 21 | 24 | Supramarginal Gyrus |
| 47 | 61 | -22 | Cerebellum |
| **-35** | **55** | **16** | **Middle Occipital Gyrus** |
| -47 | 65 | -18 | Cerebellum |
| -33 | 85 | -4 | Inferior Occipital Gyrus |
| 27 | 63 | 52 | Superior Parietal Lobule |
| -25 | 65 | 50 | Superior Parietal Lobule |
| -21 | 61 | 10 | Calcarine Gyrus |
| -47 | 9 | 44 | Precentral Gyrus |
| **-7** | **-23** | **28** | **Orbital Gyrus** |
| 41 | 7 | 40 | Precentral Gyrus |
| -37 | 47 | -22 | Cerebellum |
| -25 | -1 | 8 | Putamen |
| **-15** | **-7** | **-10** | **Superior Temporal Gyrus** |
| 49 | -5 | 14 | Inferior Frontal Gyrus |
| 41 | -29 | -4 | Inferior Frontal Gyrus |
